# Factors influencing adoption of self-monitoring of blood pressure among hypertensive patients in primary healthcare in Vietnam: a cross-sectional facility-based study

**DOI:** 10.1186/s12875-025-02871-5

**Published:** 2025-05-21

**Authors:** Nguyen Hoang Giang, Nguyen Thi Phuong Lan, Le Thi Kim Anh, Dominika Plancikova, Viera Rusnakova, Nguyen Thi Thang, Jaap A.R. Koot

**Affiliations:** 1 Public Health Department Health Strategy and Policy Institute, Ministry of Health, Hanoi, Vietnam; 2 Thai Nguyen University of Pharmacy and Medicine, Thai Nguyen, Vietnam; 3 Hanoi University of Public Health, Hanoi, Vietnam; 4 Institute for Global Health and Epidemiology, Department of Public Health, Faculty of Health Care and Social Work, Trnava University, Trnava, Slovak Republic; 5 Department of Health Sciences, University of Groningen, University Medical Center Groningen, Hanzeplein 1, Building 3217, Groningen, 9700 RB The Netherlands

**Keywords:** Self-monitoring of blood pressure, Hypertension, Primary healthcare, Vietnam, Chronic disease management, Self-care practices

## Abstract

**Background:**

Hypertension is an increasing health problem in low- and middle-income countries such as Vietnam. Self-monitoring of blood pressure (SMBP) is a crucial component of hypertension management in primary healthcare (PHC) and stimulated by healthcare providers. Yet, its adoption remains suboptimal. This study examines the application and contributing factors to adoption of SMBP among hypertensive patients in a PHC setting in Vietnam.

**Methods:**

A cross-sectional study was conducted among 989 hypertensive patients managed at commune health stations (CHSs), part of PHC, in two provinces in Vietnam. Participants were selected using systematic random sampling from CHS patient lists. Data were collected through structured interviews and analysed using descriptive statistics and multivariate logistic regression to identify factors contributing to SMBP practices.

**Results:**

The prevalence of SMBP among hypertensive patients was 43.1%. Among those who practiced SMBP, 42.3% monitored their blood pressure daily, while 57.7% measured it at least weekly. The majority (80.2%) used home sphygmomanometers, 12% relied on friends or relatives with BP monitoring devices, 7.8% went to a pharmacy of health worker’s home to measure BP. Multivariate analysis identified key factors associated with SMBP adoption. Higher educated persons were more engaged in SMBP (*p* < 0.001). Pensioners were more likely to engage in SMBP than unemployed individuals (OR = 2.2; *p* < 0.001). Higher knowledge of hypertension management (OR = 1.10; *p* < 0.001) and regular physical activity (OR = 1.54; *p* = 0.005) were also positively associated with SMBP practice. Persons living in Ninh Bing were also more likely to apply SMBP (OR = 1.58; *p* < 0.001).

**Conclusion:**

Less than half of hypertensive patients practiced SMBP, highlighting a need for targeted interventions to promote self-monitoring. Key facilitators for patients include better health knowledge, socioeconomic stability, and local healthcare service availability. On the service provision side, strategies to improve SMBP adoption should focus on increasing patient education, ensuring the affordability of BP monitoring devices, and strengthening the role of primary healthcare providers in coaching patients on SMBP. A patient-centred, community-based approach is necessary to enhance hypertension self-management and improve overall cardiovascular health outcomes in Vietnam.

**Supplementary Information:**

The online version contains supplementary material available at 10.1186/s12875-025-02871-5.

## Introduction

Hypertension is the single most important risk factor for premature death worldwide, as it is a severe chronic condition that greatly elevates the risk of heart, brain, kidney, and other diseases [1]. Hypertension is annually accounting for 235 million disability adjusted life years lost (DALY) and 10.8 million deaths [2, 3]. The prevalence of hypertension has increased dramatically in recent decades [3]. About 78% of adults with hypertension is living in low- and middle-income countries (LMICs) [4]. Despite effective prevention and treatment options, hypertension remains underdiagnosed, undertreated, and under-controlled in many countries [3]. Globally, among adults aged 30–79 years with hypertension, only 54% has been diagnosed, 42% is receiving treatment, and 21% has the hypertension under control [5]. Evidence shows that a low treatment adherence among patients with hypertension is significantly associated with several adverse cardiovascular outcomes [6–8].

In Vietnam, non-communicable diseases (NCDs) have become the main cause of morbidity and mortality, with cardiovascular diseases being the primary contributor responsible for 42% of deaths and contributing 17% of the total DALYs related to NCDs [9]. Hypertension, one of the most common chronic NCDs, affects over 26.2% of the adult population in the country. However, only 24.7% of people with hypertension were being managed at health facilities, and just one third of hypertensive patients on anti-hypertensive medication had their blood pressure under control in 2021 [10].

The best way to prevent stroke and heart disease is by managing high blood pressure effectively [3]. Self-monitoring and self-management of blood pressure can be successful in lowering blood pressure levels in patients with hypertension [11]. Extensive evidence suggests that self-monitoring of blood pressure (SMBP) is associated with a reduction of systolic blood pressure among patients with hypertension compared to periodical measurement in medical settings [12, 13]. As SMBP offers advantages over clinic measurements, several international hypertension guidelines endorse SMBP [14, 15]. It allows for multiple readings over time and in the patient’s usual environment, producing a more reliable blood pressure value that is not influenced by the “white coat” or placebo effects [16]. Extensive evidence indicates that SMBP is associated with significant reduction in blood pressure and improved hypertension control, with the greatest benefits observed when combined with other interventions [14, 17]. Incorporating SMBP alongside clinical-setting BP measurement has been shown to be cost-effective compared to clinical BP monitoring alone or standard care for individuals with hypertension. SMBP fosters patients’ involvement in their care and promotes treatment adherence [14, 17, 18]. According to a systematic review with meta-analysis, SMBP has been found to have positive effects on patients with hypertension-related comorbidities as well as those with specific conditions such as obesity [17].

Previous studies have found several factors influencing adoption of SMBP among patients with hypertension. These include age, gender, education level, income, perceived health status, presence of comorbidities, use of antihypertensive medication, access to healthcare, and social support [19]. Other factors such as patients’ beliefs about the importance of self-monitoring and their confidence in their ability to perform the task may also play a role [20]. Additionally, factors such as the availability of reliable and affordable blood pressure monitoring devices and access to information and resources on how to perform self-monitoring may impact patients’ likelihood of engaging in this practice [21]. A number of studies suggest that provider-related factors affect SMBP and highlight the importance of a supportive health system for effective hypertension self-management, such as ease of access to healthcare facilities for follow-up and guidance, and healthcare providers’ support for patients [22].

While the benefits of SMBP are well-documented in international literature, there is limited understanding of its application and influencing factors among hypertensive patients within primary health care (PHC) settings in Vietnam. Acquiring more insights is relevant because the country foresees a crucial role of the PHC system in NCD management, with the provision of regular hypertension treatment under the social health insurance programme being a core strategy for NCD control [23]. Commune health stations (CHSs) serve as the first point of care for the people in each commune, offering insured patients free regular treatment for hypertension. In addition, with a modest co-payment, insured patients can seek outpatient treatment at district hospitals or higher-level public and private health facilities. Hypertensive patients are entitled to monthly consultations at these facilities, which include blood pressure monitoring, clinical assessments, counselling, and medication prescriptions. SMBP is supposed to be part of the treatment regimen. Despite this structured care system, there is a gap in knowledge about how frequently and effectively hypertensive patients engage in SMBP. This study aims to assess the personal and health system-related factors influencing adoption of SMBP among patients with hypertension managed in the PHC facilities in urban and rural communes in two provinces in Vietnam.

## Methods

### Study design and setting

A cross-sectional survey was conducted among hypertensive patients managed by 21 purposely selected CHSs, each located in a different commune, across four districts in Ninh Binh province and three districts in Hai Phong city, Vietnam (three communes per district). Ninh Binh, a northern province in the Red River Delta, has a population of over 1.2 million, with 78.4% residing in rural areas. In this province, all selected CHSs provided outpatient services through the social health insurance programme.

Hai Phong, a nearby city in the same region, has a population of approximately 2.1 million, with 54.3% living in peri-urban areas [24]. However, in Hai Phong, six CHSs in two peri-urban districts did offer outpatient services covered by social health insurance, while three CHSs in an urban district did not provide curative services. Consequently, insured patients in these areas had to seek care at higher-level public health facilities, or from private providers.

### Sampling and data collection

In 2021, a sample of 989 patients with a confirmed diagnosis of hypertension was recruited from participating CHSs. The study team obtained lists of patients diagnosed with hypertension from each CHS. or each district, aggregated lists were created by combining patient lists from three local CHSs. Using these lists, participants were selected through systematic random sampling. Eligible participants were adults (aged 18 years and older) who had been enrolled in hypertension management at their local CHS for at least six months, were able to participate in a face-to-face interview, and provided informed consent. Trained enumerators conducted these interviews utilising structured quantitative questionnaires developed with ODK software, installed on mobile tablets. The questionnaire was developed for this survey and is available as additional file to this publication. Prior to data collection, all enumerators underwent a comprehensive training program on interview techniques, questionnaire content, and ethical considerations. A pilot test was conducted to ensure clarity and appropriateness of the questions. During data collection, supervisors regularly monitored fieldwork, reviewed collected data for completeness and consistency, and provided immediate feedback to enumerators when needed. The digital platform enabled real-time data capture and reduced entry errors, enhancing data quality.

The sample size for this study was based on the baseline survey conducted as part of an intervention to strengthen hypertension management at the primary healthcare level. A sample size calculation was performed using the formula for comparing proportions in a pre-post design, assuming an increase in intervention outcome (e.g. treatment adherence) from 49.8%, based on a previous similar study [25], to 70% after the intervention, with 5% significance and 90% power. After adjusting for a design effect, 10% non-response rate, and 25% loss to follow-up, the required sample size was estimated at 134 patients per district. Finally, 989 patients were successfully interviewed.

### Variables

#### Dependent variables

In this study, SMBP was ascertained by patient’s self-reported dichotomised questions with a yes or no response to “Do you currently self-monitor your blood pressure using a self-monitoring blood pressure device?”. The follow-up questions were asked to identify whether patients actually performed SMBP, including “How often do you measure your BP?” and “How do you measure your BP yourself?”. The SMBP status was confirmed following two criteria: (i) frequency of SMBP: daily or weekly; (ii) the place of SMBP should be a non-clinical setting, such as, at home, or at others’ people home, such as friends, or relatives.

#### Independent variables

The study’s independent variables encompassed a comprehensive range of socio-economic, demographic, and health-related topics, along with an assessment of knowledge, attitudes, and practices (KAP) related to hypertension. Socio-economic and demographic variables included age group, gender, marital status, educational attainment, occupation, and residential areas (urban or rural). The disease-related variables comprised a diverse set of topics, including comorbidities with NCDs, family history of hypertension, a diagnosis of hypertension-related complications, duration of hypertension, and self-assessment of hypertension control. For the self-assessment of hypertension control, patients were asked whether they perceived their blood pressure to be under control according to professional recommendations. Health service utilisation encompassed the type of treatment received and the healthcare facilities providing treatment for hypertension.

In terms of health coverage, variables refer to health insurance enrolment and the specific type of health insurance held by participants. The questions related to participants’ practices encompass their adherence to healthy lifestyles, including dietary habits and exercise routines, as well as the avoidance of risky behaviours such as smoking and alcohol consumption, along with medication adherence. Questions were drawn from the well-established WHO-STEPS survey questionnaires employed in the Vietnam National STEPs Survey [26]. Additionally, these questions were adapted to be consistent with the guidelines for regular management of hypertension at the primary health care level by the Vietnam Ministry of Health [27]. The questions for the knowledge and attitude components were adapted from the Ministry of Health’s guidelines for the diagnosis, treatment, and management of hypertension at the primary health care level [27]. To ensure content validity, all items were reviewed by two clinical and public health experts from local medical institutions. Additionally, the questionnaire was pilot tested with a sub-sample of patients with hypertension in a separate CHS to assess clarity, comprehension, and cultural appropriateness. Minor revisions were made based on feedback from this pilot phase prior to the full data collection. The knowledge questionnaire comprised 15 true-false questions covering specific knowledge topics. Each correct response was awarded 1 point, while incorrect responses received 0 points. The knowledge score was the sum of the 15 questions, ranging from 0 to 15 points. The attitude questionnaire for patients with hypertension included 13 items on a 5-point Likert scale. Respondents indicated their level of agreement or disagreement with each statement, reflecting their perspectives on hypertension and disease management. The attitude score was calculated as the average of the 13 responses, ranging from 1 to 5 points. The practice questions assessed key hypertension management behaviours that support blood pressure control, including non-smoking, regular exercise, adherence to a low-salt diet, management by a healthcare facility, and current use of antihypertensive medications.

### Data analysis

All statistical analyses were conducted using complete case analysis. Only participants with non-missing values for all variables included in the final regression model were retained. The proportion of missing data was minimal and did not justify the use of imputation techniques. Categorical variables were assessed for associations using the Chi-square test, while the Mann-Whitney test was applied for continuous variables displaying non-normal distributions, examining the association with the independent variables and outcomes.

Given that the outcome variable was configured in binary form, a multivariate logistic regression analysis was executed to discern the factors contributing to SMBP. Categorical independent variables featuring more than two categories underwent conversion into multiple binary dummy variables, rendering them compatible with the regression models. The multivariate logistic analysis was carried out following backward elimination approach. All candidate independent variables that were identified based on theoretical relevance and univariate analysis, were initially included in the full base model. Then, variables were sequentially removed one at a time based on statistical non-significance (*p* > 0.05), starting with the least significant. The process continued iteratively until only variables with statistically significant associations remained in the final model. Additionally, potential effect modifications in the pertinent associations were subjected to testing. The fit of the final model was assessed through the application of the goodness of fit test and the assessment of the area under the Receiver Operating Characteristic (ROC) curve. The statistical analyses were performed using the STATA/IC Version 14 software package.

## Results

### Patients’ characteristics

As illustrated in Table 1, almost 85% of the patients with hypertension (PWH) were aged 60 years and above, with males comprising 43.9% of the total. Only a quarter of the respondents had completed high school or attained higher education. Approximately one-fifth of the patients were either unemployed or not working and one-third of them were retired while 45.8% were working. Almost all patients were insured (99.8%) and one-third of them were voluntarily enrolled.

**Table 1:** Patients’ characteristics by provinces

	Ninh Binh *n* = 640		Hai Phong *n* = 349		All *n* = 989	
	*n*	%	*n*	%	*n*	%
**Demographic and socioeconomic backgrounds**						
Age groups						
- < 50 years - 50–59 years - 60–69 years - ≥ 70 years	20 93 303 224	3.1 14.5 47.3 35.0	10 33 151 155	2.9 9.5 43.3 44.4	30 126 454 379	3.0 12.7 45.9 38.3
Male patients	269	42.0	165	47.3	434	43.9
Marital status						
- Unmarried - Married - Divorced/widowed/separated	13 504 123	2.0 78.8 19.2	4 271 74	1.1 77.7 21.2	17 775 197	1.7 78.4 19.9
Highest educational level						
- Lower than primary school - Primary school - Secondary school - High school - Graduate	69 80 355 88 48	10.8 12.5 55.5 13.7 7.5	30 46 169 62 42	8.6 13.2 48.4 17.8 12.0	99 126 524 150 90	10.0 12.7 53.0 15.2 9.1
Occupation						
- Employed/Currently working - Unemployed/not working - Pensioners	319 121 200	49.8 18.9 31.3	134 91 124	38.4 26.1 35.5	453 212 324	45.8 21.4 32.8
Living in rural areas	499	78.0	242	69.3	741	74.9
**Health insurance coverage**						
Patient with health insurance	639	99.8	348	99.7	987	99.8
Health insurance types						
- Fully subsidized HI - Partly subsidized HI - Voluntary HI - Private HI	349 47 221 22	54.6 7.4 34.6 3.4	216 43 80 9	62.1 12.4 23.0 2.6	565 90 301 31	57.2 9.1 30.5 3.1

With respect to the respondents’ health status, 61.2% of the patients had been diagnosed with hypertension during the last five years, while only 14% were diagnosed over 10 years ago (Table 2). Furthermore, over half of the patients were suffering from at least one other chronic condition in addition to hypertension. It was found that 43.3% of them believed their blood pressure was adequately controlled. Additionally, 12.5% of patients had been diagnosed with a complication related to hypertension.

**Table 2:** Patients’ health status and hypertension management practice

	Ninh Binh *n* = 640		Hai Phong *n* = 349		All *n* = 989	
	*n*	%	*n*	%	*n*	%
Number of comorbidities apart from hypertension						
- Only hypertension - 1 NCD - 2 + NCDs	304 194 142	47.5 30.3 22.2	127 126 96	36.4 36.1 27.5	431 320 238	43.6 32.4 24.1
Self-assessment of hypertension under control	291	45.5	138	39.5	429	43.4
Period since the first diagnosis of hypertension						
- 1 year - 2–5 years - 6–10 years - > 10 years	103 325 145 67	16.1 50.8 22.7 10.5	40 137 100 72	11.5 39.3 28.7 20.6	143 462 245 139	14.5 46.7 24.8 14.0
Patients with complications of hypertension	70	10.9	54	15.5	124	12.5
Facilities that patients with hypertension is currently treated						
- Self-medication/no treatment - Commune health stations - District health centers - Higher-level hospitals - Private health facilities	86 413 119 17 5	13.4 64.5 18.6 2.7 0.8	129 77 95 42 6	37.0 22.1 27.2 12.0 1.7	215 490 214 59 11	21.7 49.5 21.6 6.0 1.1
No smoking	583	91.1	305	87.4	888	89.8
PWH take less than 2 + drinks and 5 days a week	614	95.9	333	95.4	947	95.8
PWH is doing regular physical exercise	450	70.3	262	75.1	712	72.0
PWH has a low-salt intake diet	280	43.8	167	47.9	447	45.2
PWH is currently taking anti-hypertensive medications	618	96.6	322	92.3	940	95.0

Approximately half (49.5%) of the patients were presently receiving treatment at their local CHS, while 28.7% were treated by higher-level facilities. 21.7%, were not under the management of any healthcare facility, and instead opted for self-medication or no treatment.

Regarding the adoption of a healthy lifestyle, most patients reported adhering to recommended practices. Specifically, a significant percentage refrained from smoking (89.8%), engaged in regular exercise (72%), and practiced moderate alcohol consumption (95.8%). Furthermore, 95% of patients were currently taking anti-hypertensive medication. However, over half of the patients (54.8%) did not maintain a low-salt intake diet.

### Practice of self-monitoring blood pressure

As shown in Table 3, it was reported that 43.1% of patients with hypertension self-monitored their blood pressure regularly. Of those who engaged in SMBP, 42.3% tracked their blood pressure daily, while 57.7% measured their blood pressure at least weekly. Regarding the circumstance of SMBP, 80.2% used home sphygmomanometers and 12% sought the support from their friends or relatives who had home-based sphygmomanometers.

**Table 3:** SMBP among patient with hypertension

	Ninh Binh *n* = 640		Hai Phong *n* = 349		All *n* = 989	
	*n*	%	*n*	%	*n*	%
**Patients who are self-monitoring their blood pressure**	295	46.1	131	37.5	426	43.1
**Locations where patients self-monitored their blood pressure**						
- Patients’ home - A friend/relative’s home - A local health staff’s home - A nearby health facility or pharmacy	234 41 10 9	79.6 13.9 3.4 3.1	107 10 1 13	81.7 7.6 0.8 9.9	341 51 11 22	80.2 12.0 2.6 5.2
**Frequency of SMBP**						
- Daily - Weekly	123 172	41.7 58.3	57 74	43.5 56.5	180 246	42.3 57.7

### The factors influencing the adoption of SMBP

As presented in Table 4, female patients, those living with a spouse or partner, individuals with higher levels of education, and those with income from employment or pensions were more likely to engage in self-monitoring of their blood pressure when compared to their counterparts with different sociodemographic profiles. Patients residing in Ninh Binh exhibited a notably higher prevalence of SMBP adoption compared to those living in Hai Phong. In addition, patients who reported higher levels of physical activity, adhered to a low-salt diet, and currently treated by a health facility seems to more be associated with SMBP practice. Patients who demonstrated better knowledge and a positive attitude towards managing their hypertension were more likely to engage in SMBP.

**Table 4:** The practice of SMBP by patient’s characteristics

	Self-monitoring				*P*-values*
	No		Yes		
	*n*	%	*n*	%	
**Age groups**					
- < 60 years - 60–69 years - ≥ 70 years	81 270 212	51.9 59.5 55.9	75 184 167	48.1 40.5 44.1	0.230
**Gender**					
- Male - Female	336 227	60.5 52.3	219 207	39.5 47.7	**0.009**
**Marital status**					
- Others - Married	136 427	63.6 55.1	78 348	36.5 44.9	**0.027**
**Highest educational level**					
- Primary school and lower - Secondary school - High school - Graduate	154 289 78 42	68.4 55.2 52.0 46.7	71 235 72 48	31.6 44.8 48.0 53.3	**< 0.001**
**Occupation**					
- Currently working - Pensioners - Unemployed/not working	263 91 209	58.1 42.9 64.5	190 121 115	41.9 57.1 35.5	**< 0.001**
**Province of residence**					
- Hai Phong - Ninh Binh	218 345	62.5 53.9	131 295	37.5 46.1	**0.009**
**Rural/urban**					
- Urban - Rural	131 432	52.8 58.3	117 309	47.2 41.7	0.132
**Health insurance types**					
- Fully subsidized HI - Partly subsidized HI - Voluntary HI	315 57 190	55.8 63.3 57.2	250 33 142	44.2 36.7 42.8	0.399
Self-assessment of HTN under control					
- No - Yes	333 230	59.5 53.6	227 199	40.5 46.4	0.066
Time period since the first diagnosis					
- 1 year - 2–5 years - 6–10 years - > 10 years	90 205 189 79	62.9 58.1 53.4 56.8	53 148 165 60	37.1 41.9 46.6 43.2	0.251
Any diagnosed complications of HTN					
- No - Yes	68 495	54.8 57.2	56 370	45.2 42.8	0.616
Number of comorbidities apart from HTN					
- Only HTN - 1 NCD - 2 + NCDs	261 179 123	60.6 55.9 51.7	170 141 115	39.4 44.1 48.3	0.077
Smoking					
- No - Yes	501 62	56.4 61.4	387 39	43.6 38.6	0.339
Take less than two standard drinks five days a week					
- No - Yes	539 24	56.9 57.1	408 18	43.1 42.9	0.977
Regular physical exercise					
- No - Yes	182 381	65.7 53.5	95 331	34.3 46.5	**0.001**
Low-salt intake diet (less than 5 g per day)?					
- No - Yes	329 234	60.7 52.3	213 213	39.3 47.7	**0.008**
Currently treated at a health facility					
- No - Yes	144 419	67.0 54.1	71 355	33.0 45.9	**0.001**
Facilities that PWH is currently treated					
- Self-medication/no treatment - Commune health station - District health centres - Higher-level hospitals	144 268 117 34	67.0 54.7 54.7 48.6	71 222 97 36	33.0 45.3 45.3 51.4	**0.006**
Mean total knowledge score (SD)	10.85 (3.55)		11.89 (2.60)		**< 0.001**
Mean attitude score (SD)	3.55 (0.61)		3.63 (0.58)		**0.016**

* Note: Categorical variables were applied the Chi-square test and Mann-Whitney test was applied for continuous variables displaying non-normal distributions

The results of univariate logistic regression analysis are represented in Table 5. In the final model, the multivariable logistic regression analysis revealed that occupation, knowledge of hypertension management, regular physical activities, and province of residence were significant factors associated with SMBP practice among patients with hypertension (Table 6). Pensioners were 2.23 times (OR = 2.23; *p* < 0.001) more likely to adopt SMBP compared to those who were unemployed or not working. Patients who demonstrated a better understanding of hypertension management had a higher odds ratio (OR = 1.10; *p* < 0.001) of engaging in SMBP practices. Engaging in regular physical activities was another significant factor associated with SMBP adoption. Patients who reported being physically active had 1.54 times higher odds (OR = 1.54; *p* = 0.005) of practicing SMBP. Finally, patients who resided in Ninh Binh were 1.58 times (OR = 1.58; *p* < 0.001) more likely to SMBP compared to those living in Hai Phong.

**Table 5:** The results of simple logistic regression analysis on patient’s characteristics

Variables	Crude OR	SE	*p*	95% CI	
**Age groups**					
- < 60 years - 60–69 years - ≥ 70 years	- 0.74 0.85	- 0.13 0.16	0.100 0.397	0.51 0.59	1.06 1.24
**Gender**					
- Female - Male	- 1.40	- 0.18	- 0.010	- 1.09	- 1.80
**Marital status**					
- Others - Married	- 1.42	- 0.23	- 0.027	- 1.04	- 1.94
**Highest educational level**					
- Primary school and lower - Secondary school - High school - Graduate	- 1.76 2.00 2.48	- 0.30 0.44 0.63	- 0.001 0.001 0.000	- 1.27 1.31 1.50	- 2.45 3.07 4.09
**Occupation**					
- Unemployed/not working - Currently working - Pensioners	- 1.31 2.42	- 0.20 0.44	- 0.070 0.000	- 0.98 1.70	- 1.76 3.45
**Province of residence**					
- Hai Phong - Ninh Binh	- 1.42	- 0.19	- 0.010	- 1.09	- 1.86
**Rural/urban**					
- Urban - Rural	- 0.80	- 0.12	- 0.132	- 0.60	- 1.07
**Health insurance types**					
- Voluntary HI - Fully subsidized HI - Partly subsidized HI	- 1.06 0.77	- 0.15 0.19	- 0.667 0.298	- 0.81 0.48	- 1.40 1.25
Self-assessment of HTN under control					
- No - Yes	- 1.27	- 0.16	- 0.066	- 0.98	- 1.64
Time period since the first diagnosis					
- 1 year - 2–5 years - 6–10 years - > 10 years	- 1.23 1.48 1.29	- 0.25 0.30 0.31	- 0.318 0.053 0.296	- 0.821 0.995 0.800	- 1.829 2.208 2.079
Any diagnosed complications of HTN					
- No - Yes	- 0.91	- 0.18	- 0.616	- 0.622	- 1.325
Number of comorbidities apart from HTN					
- Only HTN - 1 NCD - 2 + NCDs	- 1.21 1.44	- 0.18 0.23	- 0.204 0.026	- 0.90 1.04	- 1.62 1.98
Smoking					
- No - Yes	- 0.814	- 0.18	- 0.340	- 0.534	- 1.242
Take less than two standard drinks five days a week					
- No - Yes	- 0.99	- 0.32	- 0.977	- 0.531	- 1.850
Regular physical exercise					
- No - Yes	- 1.66	- 0.24	- 0.001	- 1.247	- 2.221
Low-salt intake diet					
- No - Yes	- 1.41	- 0.18	- 0.008	- 1.091	- 1.911
Currently treated at a health facility					
- No - Yes	- 1.718	- 0.28	- 0.001	- 1.251	- 2.360
Facilities that PWH is currently treated					
- Self-medication/no treatment - Commune health station - District health centres - Higher-level hospitals	- 1.68 1.68 2.15	- 0.28 0.34 0.60	- 0.002 0.009 0.006	- 1.201 1.367 1.241	- 2.349 2.487 3.715
Mean total knowledge score	1.11	0.02	0.000	1.068	1.166
Mean attitude score	1.26	0.14	0.030	1.023	1.562

**Table 6:** The multivariate logistic analysis determining individual factors associated with SMBP practice among hypertensive patients

Variables	Adjusted-OR	SE	*p*-values	95% CI	
Occupation					
- Unemployed/not working - Employed/Currently working - Pensioners	1 1.29 2.23	- 0.20 0.41	- 0.098 < 0.001	- 0.95 1.55	- 1.74 3.20
Knowledge about hypertension	1.10	0.03	< 0.001	1.05	1.15
Regular physical exercise					
- No - Yes	1 1.54	- 2.83	- 0.005	1.14	2.08
Province of residence					
- Ninh Binh - Hai Phong	1.58 1	3.24 -	< 0.001	1.20	2.09

## Discussion

To the best of our knowledge, this study represents one of the pioneering efforts to evaluate both the extent and influencing factors on adoption of SMBP among individuals with hypertension in Vietnam. The findings emphasise that socioeconomic factors, specifically occupation status, patients’ understanding of hypertension management, adherence to a healthy lifestyle, and residential location, are significant predictors for the practice of SMBP among hypertensive patients. These findings highlight the importance of targeted interventions to promote SMBP, considering sociodemographic and lifestyle factors to enhance hypertension management and control.

The study revealed that 43.1% of patients with hypertension regularly self-monitored their blood pressure, with 42.3% doing so daily and 57.7% at least weekly. Previous studies show a wide variability of SMBP in various countries. Globally, engagement in SMBP among patients with hypertension ranged from 9 to 75% [28–34]. SMBP was higher in Italy (75%) and Pakistan (61.7%) [30, 34], more or less the same in Canada (45.9%) and Oman (40%), but much lower in Ethiopia (8.9%), Singapore (24%), USA (25%) and UK (30%).

SMBP is strongly influenced by cultural, healthcare, and regional factors. It is therefore necessary to engage in context-specific healthcare interventions to enhance SMBP practices and hypertension management.

Patient-related factors are crucial in SMBP. Almost 85% of patients with hypertension enrolled in our study in local CHSs and district hospitals were older people. This can partially be explained because older patients are more likely to suffer from NCDs, particularly hypertension, than their younger counterparts [35]. In addition, older people prefer to seek care at the local health facilities, given their limited access to higher-level health facilities due to financial and transportation constraints [35].

The findings from our study in Vietnam illuminate a compelling association between socioeconomic status and SMBP. In our sample, over 75% of patients had an income from their pension or employment. Notably, pensioners were 2.2 times more likely to embrace SMBP compared to unemployed individuals. Similarly, currently employed individuals demonstrated a 1.3-fold higher likelihood of SMBP adoption. In the current study, over 80% of patients who engaged SMBP used their automated sphygmomanometers at home. Better-off patients were more likely to afford a home-based BP monitoring device than their socioeconomically disadvantaged counterparts. These findings align with studies highlighting the positive correlation between socioeconomic status, particularly employment, and health-seeking behaviours [36–39]. Comparisons with other studies in the literature may further elucidate these dynamics, providing a nuanced understanding of how socioeconomic disparities influence the adoption of SMBP across diverse populations and contexts.

The multivariable regression analysis indicated that patients with a more profound understanding of hypertension management exhibited a significantly higher odds ratio of adopting SMBP, highlighting the pivotal role of knowledge in fostering proactive health behaviours. This finding is in line with a growing body of literature emphasising the importance of health literacy in NCD management, particularly self-management of hypertension, in Vietnam [40, 41] and other LMICs [38, 39, 42–44]. Studies across diverse settings consistently underscore the positive impact of health education and literacy programmes on empowering individuals to actively participate in the management of chronic conditions [22, 45–49]. Still, different contexts influence health behaviours [47, 49]. Recognising and addressing these contextual differences can improve tailored interventions aiming at improving health literacy and subsequently enhancing the adoption of SMBP practices among patients with hypertension in Vietnam and comparable LMICs. Future research into culturally sensitive interventions in diverse populations might enhance acceptance of SMBP.

The current study identified a positive association between an active lifestyle and proactive disease management practice. Patients who reported engaging in regular physical activities exhibited a significant 1.5 times higher odds of practicing SMBP. This finding supports the existing literature emphasising the prioritization of increased physical activity as a key intervention strategy for self-managing hypertension in Vietnam [45, 50, 51], and LMICs [47, 52–58]. It suggests the need for public health campaigns advocating for increased physical activity and self-monitoring practices.

There are also health services-related factors important in SMBP. Hypertension care under the health insurance scheme contributes to SMBP. Patients residing in Ninh Binh exhibited a significant 1.6 times higher chance of practicing SMBP compared to their counterparts in Hai Phong. In Ninh Binh, a majority of CHSs offered curative services under the social health insurance scheme, leading to 64.5% of patients seeking hypertension treatment at the lowest level of PHC (as shown in Table 2). Conversely, health insurance-based services were not widely available at the CHSs in urban settings of Hai Phong city. Our study’s finding may illustrate how the availability of health insurance-based services at the CHSs in Ninh Binh may contribute to the increased likelihood of SMBP practice among patients with hypertension.

The level of health facility for hypertension treatment can be a factor that influence SMBP. Our study showed that 55.6% patients received counselling services at the CHSs, while only 46.2% at the district hospitals (as reported in Appendix 4). Another study showed that doctors at district hospitals spent less time per consultation and asked fewer questions per patient compared to doctors at CHSs [59]. One possible explanation was that doctors in district hospitals face a heavier caseload and thus have less time available per patient [59]. SMBP, when integrated into patient-healthcare professionals’ interactions, is more likely to be effective than a single SMBP intervention [47]. The variations in local healthcare settings must be considered when designing targeted interventions to promote disease self-management practices among patients with chronic NCDs in Vietnam.

Several limitations were acknowledged in the study. The most important is that the outcome measures were self-reported. The use of self-reports for SMBP practice is common, and, in this study, respondents were carefully assessed with a set of questions. Similar methods have been used in other studies [37, 39]. Furthermore, it is crucial to recognise that the predominant representation of older patients in the study sample may impose constraints on the generalisability of the findings to the broader population with hypertension in Vietnam. Although hypertension is commonly associated with older age, the skewed age distribution should be considered when interpreting the study’s applicability to a more age-diverse population.

## Conclusion

Our study provides important insights into the adoption and contributing factors of SMBP practices among hypertensive patients in Vietnam, an LMIC context where hypertension self-management remains suboptimal. With less than half of patients engaging in SMBP, there is a clear need to scale up this practice to improve hypertension control. Our findings underscore the role of knowledge, socioeconomic status, and healthcare service availability in facilitating SMBP, suggesting that interventions should prioritize vulnerable populations with limited resources. Efforts should focus on making SMBP devices more affordable through subsidies or market-based strategies, improving accessibility through community health programmes, and strengthening the capacity of primary healthcare providers to educate and support patients in self-monitoring. A comprehensive, patient-centred approach that addresses financial and knowledge barriers can enhance SMBP adoption, ultimately improving hypertension management and reducing the burden of cardiovascular diseases in Vietnam. Health insurance schemes which make hypertension management possible at CHS level may contribute to stimulating SMBP.

## Electronic supplementary material

Below is the link to the electronic supplementary material.

Supplementary Material 1

Supplementary Material 2

## Data Availability

The questionnaire used in this research was developed for this purpose, and is available as additional file to this publication. Data were not used for any other publication. Additional data tables are available in additional files to this article. The raw data on which this publication is based, are available from the corresponding author on request.
