# Supplementary material for: Factors influencing adoption of self-monitoring of blood pressure among hypertensive patients in primary healthcare in Vietnam: a cross-sectional facility-based study
Source: BMC Prim Care. 2025 May 21;26:180. doi: 10.1186/s12875-025-02871-5 (PMC12093837; doi:10.1186/s12875-025-02871-5)
Supplement: Supplementary file 1 — Supplementary Material 1 [file 12875_2025_2871_MOESM1_ESM.docx]

Addditional files SMBP Vietnam

**APPENDIX**

Appendix 1: Patients’ knowledge about hypertension (Appendix)

| **Correct answers (%)** | **Ninh Binh**  **n=640** | | **Hai Phong**  **n=349** | | **All**  **n=989** | |
| --- | --- | --- | --- | --- | --- | --- |
|  | n | % | n | % | n | % |
| Correct BP reading to confirm hypertension | 418 | 65.3 | 223 | 63.9 | 641 | 64.8 |
| Hypertension usually has no symptoms | 189 | 29.5 | 103 | 29.5 | 292 | 29.5 |
| High salt intake is a risk factor for hypertension | 522 | 81.6 | 294 | 84.2 | 816 | 82.5 |
| Smoking is a risk factor for hypertension | 499 | 78.0 | 295 | 84.5 | 794 | 80.3 |
| Being overweight/obese is a risk factor for hypertension | 513 | 80.2 | 295 | 84.5 | 808 | 81.7 |
| Lack of physical activity is a risk factor for hypertension | 536 | 83.8 | 292 | 83.7 | 828 | 83.7 |
| Hypertension is caused by aging, so it is unnecessary to get treatment | 489 | 76.4 | 277 | 79.4 | 766 | 77.5 |
| Hypertension is completely treated | 349 | 54.5 | 217 | 62.2 | 566 | 57.2 |
| People with hypertension should take medication only when the blood pressure increases | 315 | 49.2 | 193 | 55.3 | 508 | 51.4 |
| People with hypertension (PWH) must take their anti-hypertensive medicine for their whole life | 575 | 89.8 | 304 | 87.1 | 879 | 88.9 |
| PWH have to attend regular medical appointments and receive anti-hypertensive medications | 605 | 94.5 | 330 | 94.6 | 935 | 94.5 |
| Untreated hypertension can cause heart diseases | 556 | 86.9 | 332 | 92.3 | 878 | 88.8 |
| Untreated hypertension can cause stroke | 585 | 91.4 | 318 | 91.1 | 903 | 91.3 |
| Untreated hypertension can cause kidney failure | 487 | 76.1 | 281 | 80.5 | 768 | 77.7 |
| Untreated hypertension can cause eye complications | 511 | 79.8 | 282 | 80.8 | 793 | 80.2 |
| **Mean total knowledge score (SD)** | **11.2 (3.22)** | | **11.5 (3.19)** | | **11.3 (3.21)** | |

Appendix 2: Patients’ attitude toward hypertension management (Appendix)

| **Likert-5 score (mean, SD)** | **Ninh Binh**  **n=640** | | **Hai Phong**  **n=349** | | | **All**  **n=989** | | |
| --- | --- | --- | --- | --- | --- | --- | --- | --- |
|  | mean | SD | mean | SD | mean | | SD |  |
| Hypertension is a dangerous disease | 4.50 | 0.51 | 4.32 | 0.83 | 4.44 | | 0.68 |  |
| Untreated hypertension can lead to many severe health complications | 4.39 | 0.60 | 4.22 | 0.86 | 4.33 | | 0.71 |  |
| PWH must follow continuous and lifelong treatment | 4.31 | 0.64 | 4.17 | 0.89 | 4.26 | | 0.74 |  |
| When BP is stable, the patient can self-reduce the dose or stop taking the medicines (reserve score) | 2.83 | 1.30 | 3.01 | 1.37 | 2.89 | | 1.33 |  |
| PWH should take medications as prescribed | 4.32 | 0.62 | 4.13 | 0.86 | 4.26 | | 0.72 |  |
| While taking medications as prescribed, PWH doesn’t have to change lifestyle behaviours (reserve score) | 2.92 | 1.32 | 3.08 | 1.37 | 2.98 | | 1.34 |  |
| Regular exercise can support controlling blood pressure | 4.22 | 0.62 | 4.07 | 0.84 | 4.16 | | 0.71 |  |
| Low salt intake can support controlling blood pressure | 4.19 | 0.68 | 4.07 | 0.84 | 4.15 | | 0.75 |  |
| PWH can smoke if BP is under control (reserve score) | 3.04 | 1.33 | 3.28 | 1.40 | 3.12 | | 1.36 |  |
| Limiting alcohol consumption can support controlling blood pressure | 3.99 | 0.91 | 4.03 | 0.94 | 4.00 | | 0.92 |  |
| Although blood pressure is controlled, PWH still has to attend the periodic medical appointment | 4.22 | 0.66 | 4.12 | 0.85 | 4.18 | | 0.73 |  |
| PWH should self-monitor my blood pressure regularly | 4.12 | 0.71 | 4.06 | 0.84 | 4.10 | | 0.76 |  |
| Both PWHs and doctors have important roles in controlling BP | 4.24 | 0.59 | 4.09 | 0.83 | 4.19 | | 0.69 |  |
| **Mean attitude score (SD)** | **3.60** | **0.54** | **3.56** | **0.68** | **3.59** | | **0.60** |  |

Appendix 3: Patients’ practice on hypertension management (Appendix)

|  | **Ninh Binh**  **n=640** | | **Hai Phong**  **n=349** | | **All**  **n=989** | |
| --- | --- | --- | --- | --- | --- | --- |
|  | n | % | n | % | n | % |
| No smoking | 91.1 | 583 | 87.4 | 305 | 888 | 89.8 |
| PWH take less than 2+ drinks and 5 days a week | 614 | 95.9 | 333 | 95.4 | 947 | 95.7 |
| PWH is doing regular physical exercise (5 days/week) | 450 | 70.3 | 262 | 75.1 | 712 | 72.0 |
| PWH has a low-salt intake diet | 280 | 56.3 | 182 | 52.2 | 542 | 54.8 |
| PWH is currently managed by a health facility | 555 | 86.7 | 222 | 63.6 | 777 | 78.6 |
| PWH is currently taking anti-hypertensive medications | 618 | 97.5 | 322 | 98.2 | 940 | 97.7 |
| Self-monitoring of BP (daily/weekly) | 295 | 46.1 | 131 | 37.5 | 426 | 43.1 |

Appendix 4. Health services that patients with hypertension received in the last visit to PHC facilities

| **Services that patients received in the last visit to PHC facilities** | **Commune Health station**  **(n=437)** | | **District hospital**  **(n=233)** | |
| --- | --- | --- | --- | --- |
|  | **n** | **%** | **n** | **%** |
| Health services received at the local CHS   - Anthropometric measures - Blood pressure measurement - Asking for symptoms/risk factors - Counselling - Medication supply - Tests (blood, urine) | 41  419  185  243  422  6 | 9.4  95.9  42.3  55.6  96.6  1.4 | 19  210  79  103  207  127 | 8.5  94.2  35.4  46.2  92.8  56.9 |
