# Supplementary material for: Factors influencing adoption of self-monitoring of blood pressure among hypertensive patients in primary healthcare in Vietnam: a cross-sectional facility-based study
Source: BMC Prim Care. 2025 May 21;26:180. doi: 10.1186/s12875-025-02871-5 (PMC12093837; doi:10.1186/s12875-025-02871-5)
Supplement: Supplementary file 2 — Supplementary Material 2 [file 12875_2025_2871_MOESM2_ESM.pdf]

SUNI-SEA PATIENT SURVEY

HYPERTENSION VIETNAM

Variable code	Variable label	Variable type	Variable value label
tinh	Province	Categorical	1. Ninh Binh<br>2. Hai Phong
huyen	District	Categorical	1. Gia Vien<br>2. Tam Diep<br>3. Ninh Binh city<br>4. Yen Mo<br>5. An Duong<br>6. Le Chan<br>7. Tien Lang
csyt	Name of commune health station	Categorical	1. Gia Hung<br>2. Gia Trung<br>3. Lien Son<br>4. Tay Son<br>5. Yen Binh<br>6. Yen Son<br>7. Ninh Khanh<br>8. Ninh Phong<br>9. Ninh Nhat<br>10. Yen Dong<br>11. Yen Nhan<br>12. Yen Phong<br>13. An Bien<br>14. An Dong<br>15. An Duong<br>16. An Hoa<br>17. An Hong<br>18. An Hung<br>19. Bac Hung<br>20. Bac Son<br>21. Bach Dang<br>22. Cap Tien<br>23. Cat Dai<br>24. Dai Ban<br>25. Dai Thang<br>26. Dang Cuong<br>27. Doan Lap<br>28. Dong Hai<br>29. Dong Hung

Variable code	Variable label	Variable type	Variable value label
			30. Dong Thai<br>31. Du Hang<br>32. Du Hang Kenh<br>33. Hang Kenh<br>34. Ho Nam<br>35. Hong Phong<br>36. Hong Thai<br>37. Hung Thang<br>38. Kenh Duong<br>39. Khoi Nghia<br>40. Kien Thiet<br>41. Lam Son<br>42. Le Loi<br>43. Le Thien<br>44. Nam Hung<br>45. Nam Son<br>46. Nghia Xa<br>47. Niem Nghia<br>48. Quang Phuc<br>49. Quoc Tuan<br>50. Quyet Tien<br>51. Tan Tien<br>52. Tay Hung<br>53. Tien Lang<br>54. Tien Cuong<br>55. Tien Hung<br>56. Tien Minh<br>57. Tien Thang<br>58. Tien Thanh<br>59. Tien Tien<br>60. Toan Thang<br>61. Trai Cau<br>62. Tran Nguyen Han<br>63. An Duong<br>64. Tu Cuong<br>65. Vinh Niem<br>66. Vinh Quang
dtv	Interviewer	Text	
ma_bn	Patient number	Continuous	
date	Date of interview	Text	
patient_id	Patient ID	Continuous	
**A. GENERAL INFORMATION OF PATIENT**			
a1	Age	Continuous	
a2	Sex	Dichotomous	1. Male<br>2. Female

Variable code	Variable label	Variable type	Variable value label
a3	Ethnicity	Dichotomous	1. Kinh<br>2. Other, specify
a3_khac	Ethnicity (other, specify)	Text	
a4	Marital status	Categorical	1. Single<br>2. Married<br>3. Separated/Divorced/ Widowed<br>4. Other, specify
a4_khac	Marital status (other, specify)	Text	
a5	Education	Categorical	1. Not going to school/Not finished primary school<br>2. Primary school<br>3. Secondary school<br>4. High school<br>5. Intermediate/College/University<br>6. Postgraduate
a6	Job	Categorical	1. Agriculture/ Forestry /Fisheries<br>2. Salary work<br>3. Services, business<br>4. Freelance<br>5. Retirement<br>6. Elderly<br>7. Go to school<br>8. Housework<br>9. Unemployed<br>10. Other, specify
a6_khac	Job (other, specify)	Text	
a7	Average monthly income	Continuous	
a8	Average family monthly income	Continuous	
a9	Household economic status	Categorical	1. Poor household<br>2. Near-poor household<br>3. Other
a10	People who are living with	Categorical	1. Alone<br>2. Wife/husband<br>3. Children<br>4. Grandchild<br>5. Brother/Sister<br>6. Other, specify
a10_khac	People who are living with (other, specify)	Text	
a11	Number of people who are aged 18 years or older	Continuous	
a12	Health insurance status	Dichotomous	1. Yes<br>2. No
a13	Types of health insurance card	Categorical	1. The insured groups fully subsidized by state budget

Variable code	Variable label	Variable type	Variable value label
			2. The insured group funded by social insurance agency<br>3. The insured group partially subsidized by state budget<br>4. Household insurance<br>5. Private health insurance<br>6. Other, specify
a13_khac	Types of health insurance card (other, specify)	Text	
a14	Registered facility for health insurance entitlement	Categorical	1. Commune health station<br>2. District health center<br>3. Provincial hospital<br>4. Private health facility<br>5. Other, specify
a14_khac	Registered facility for health insurance entitlement (other, specify)	Text	
a15	Distance from home to CHS	Continuous	
a16	Distance from home to DHC	Continuous	
a17	Means of transport to CHS	Categorical	1. Walk<br>2. Bicycle<br>3. Electric bicycle<br>4. Taxi<br>5. Private car<br>6. Public bus<br>7. Motorcycle<br>8. Go with other person
a17a	Visit CHS by yourself	Dichotomous	1. Yes<br>2. No
a17b	Average monthly income of the person who take you to CHS	Continuous	
a18	Means of transport to go to DHC	Categorical	1. Walk<br>2. Bicycle<br>3. Electric bicycle<br>4. Taxi<br>5. Private car<br>6. Public bus<br>7. Motorcycle<br>8. Go with other person
a18a	Visit DHC by yourself	Dichotomous	1. Yes<br>2. No
a18b	Average monthly income of the person who take you to DHC	Continuous	
**B. INFORMATION OF HYPERTENSION**			
b1	Ever diagnosed with hypertension	Dichotomous	1. Yes

Variable code	Variable label	Variable type	Variable value label
			2. No
b2	Health facility where you had initial diagnosis of hypertension	Categorical	1. Commune health station<br>2. District health center<br>3. Provincial hospital<br>4. Private health facility<br>5. Other, specify
b2_khac	Health facility where you had initial diagnosis of hypertension (other, specify)	Text	
b3	Remember time for first diagnosis of hypertension	Dichotomous	1. Yes<br>2. No
b3a	Month/year for first diagnosis of hypertension	Text	
b3b	Number of years since first diagnosis	Continuous	
b4	Circumstance to find out hypertension	Categorical	1. Regular health checkup<br>2. Screening event for hypertension<br>3. Medical examination for another condition<br>4. Other, specify
b4_khac	Circumstance to find out hypertension (other, specify)	Text	
b5	Remember the last time when blood pressure was measured	Dichotomous	1. Do not remember<br>2. Remember
b5a	How many days since the last time when your blood pressure was measured	Continuous	
b6	Remember your last blood pressure reading	Dichotomous	1. Yes<br>2. No
b6a	Systolic mmHg	Continuous	
b6b	Diastolic mmHg	Continuous	
b7	Other NCDs	Categorical	1. Diabetes<br>2. Coronary artery disease<br>3. Myocardial infarction<br>4. Stroke<br>5. Heart failure<br>6. Angina pectoris<br>7. Dyslipidemia<br>8. Chronic kidney disease<br>9. Retinopathy<br>10. Asthma/ Chronic Obstructive Pulmonary Disease<br>11. Arthritis<br>12. Nothing else

Variable code	Variable label	Variable type	Variable value label
b8	Remember time of the first diagnosis of diabetes	Dichotomous	1. Do not remember<br>2. Remember
b8a	Number of months since first diagnosis	Continuous	
b8b	Number of years since first diagnosis	Continuous	
b9	Blood glucose level in the last reading	Categorical	1. Do not remember<br>2. Reach target blood glucose level<br>3. Do not reach target blood glucose level
b10	Health facility where get diabetes treatment	Categorical	1. No treatment at facility<br>2. Commune health station<br>3. District health center<br>4. Provincial hospital<br>5. Private health facility<br>6. Other, specify
b10_khac	Facility where get diabetes treatment (other, specify)	Text	
b11	Buy diabetes medicine without prescription	Dichotomous	1. Yes<br>2. No
b12	Average number of days in a month to taking diabetes medicine in last 3 months	Continuous	
b13	Complications of hypertension	Categorical	1. No complication<br>2. Heart complications (myocardial infarction, heart failure)<br>3. Brain complications (stroke, cerebral hemorrhage)<br>4. Kidney complications (kidney failure, haematuria)<br>5. Eye complications (retina oedema, retinal haemorrhage)<br>6. Other, specify
b13_khac	Complications of hypertension (other, specify)	Text	
b14	Family members have hypertension	Categorical	1. Nobody<br>2. Wife/Husband<br>3. Children<br>4. Brother/Sister<br>5. Parents<br>6. Other, specify
b14_khac	Family members have hypertension (other, specify)	Text	

Variable code	Variable label	Variable type	Variable value label
b15	Whether your blood pressure is under control	Dichotomous	1. Yes<br>2. No
**C. KNOWLEDGE, ATTITUDE AND PRACTICE OF HYPERTENSION TREATMENT**			
**C1. KNOWLEDGE**			
c1	Hypertension is when maximum blood pressure is ≥ 140mmHg and/or minimum blood pressure is ≥ 90mmHg	Categorical	1. True<br>2. False<br>3. Unknown
c2	Hypertension usually has no symptoms	Categorical	1. True<br>2. False<br>3. Unknown
c3	High salt intake can cause hypertension	Categorical	1. True<br>2. False<br>3. Unknown
c4	Smoking can cause hypertension	Categorical	1. True<br>2. False<br>3. Unknown
c5	Being overweight and obese can cause hypertension	Categorical	1. True<br>2. False<br>3. Unknown
c6	Lack of physical activity can cause hypertension	Categorical	1. True<br>2. False<br>3. Unknown
c7	Hypertension is caused by ageing, so it is unnecessary to have treatment	Categorical	1. True<br>2. False<br>3. Unknown
c8	Hypertension can be completely cured	Categorical	1. True<br>2. False<br>3. Unknown
c9	Patient should take their medicine only when the blood pressure increases	Categorical	1. True<br>2. False<br>3. Unknown
c10	Patient must take their medicine for the whole life	Categorical	1. True<br>2. False<br>3. Unknown
c11	Patient have to attend regular medical appointments and receive	Categorical	1. True<br>2. False

Variable code	Variable label	Variable type	Variable value label
	long-term anti-hypertensive medications		3. Unknown
c12	If hypertension is not treated, it can cause cardiovascular disease	Categorical	1. True<br>2. False<br>3. Unknown
c13	If hypertension is not treated, it can cause stroke	Categorical	1. True<br>2. False<br>3. Unknown
c14	If hypertension is not treated, it can cause kidney failure	Categorical	1. True<br>2. False<br>3. Unknown
c15	If hypertension is not treated, it can cause eye complications	Categorical	1. True<br>2. False<br>3. Unknown
**C2. ATTITUDE**			
c16	Hypertension is a dangerous disease	Categorical	1. Strongly disagree<br>2. Disagree<br>3. Neutral<br>4. Agree<br>5. Strongly agree
c17	Hypertension can lead to many severe health problems if patients are not treated promptly	Categorical	1. Strongly disagree<br>2. Disagree<br>3. Neutral<br>4. Agree<br>5. Strongly agree
c18	Patients must follow continuous and lifelong treatment	Categorical	1. Strongly disagree<br>2. Disagree<br>3. Neutral<br>4. Agree<br>5. Strongly agree
c19	When the blood pressure is stable, patients can self-reduce the dose or stop taking the medicines	Categorical	1. Strongly disagree<br>2. Disagree<br>3. Neutral<br>4. Agree<br>5. Strongly agree
c20	Patients have to take medicine as prescription	Categorical	1. Strongly disagree<br>2. Disagree<br>3. Neutral<br>4. Agree

Variable code	Variable label	Variable type	Variable value label
			5. Strongly agree
c21	While taking anti-hypertensive drugs regularly, patients do not have to change lifestyle behaviors	Categorical	1. Strongly disagree<br>2. Disagree<br>3. Neutral<br>4. Agree<br>5. Strongly agree
c22	Doing regular exercise helps controlling blood pressure	Categorical	1. Strongly disagree<br>2. Disagree<br>3. Neutral<br>4. Agree<br>5. Strongly agree
c23	A low salt intake helps controlling blood pressure	Categorical	1. Strongly disagree<br>2. Disagree<br>3. Neutral<br>4. Agree<br>5. Strongly agree
c24	Patients can still smoke if blood pressure levels remain stable	Categorical	1. Strongly disagree<br>2. Disagree<br>3. Neutral<br>4. Agree<br>5. Strongly agree
c25	It is important to reduce or limit alcohol consumption	Categorical	1. Strongly disagree<br>2. Disagree<br>3. Neutral<br>4. Agree<br>5. Strongly agree
c26	Given blood pressure is controlled, patients still have to attend the periodical medical appointment	Categorical	1. Strongly disagree<br>2. Disagree<br>3. Neutral<br>4. Agree<br>5. Strongly agree
c27	Patients should self-monitor their blood pressure	Categorical	1. Strongly disagree<br>2. Disagree<br>3. Neutral<br>4. Agree<br>5. Strongly agree

Variable code	Variable label	Variable type	Variable value label
c28	Both patients and physicians have important role in controlling the blood pressure	Categorical	1. Strongly disagree<br>2. Disagree<br>3. Neutral<br>4. Agree<br>5. Strongly agree
**C3. PRACTICE**			
c29	Currently smoked/inhaled/chew any kind of tobacco, pipe tobacco, cigar, or pipe	Categorical	1. No<br>2. Yes, tobacco<br>3. Yes, pipe tobacco<br>4. Yes, betel
c29_khac	Smoked/inhaled/chew any kind of tobacco, pipe tobacco, cigar, or pipe (other, specify)	Text	
c30	Frequency of smoking	Categorical	1. Every day<br>2. Every week<br>3. Every month
c31	Number of cigarettes on average	Continuous	
c32	Currently drinking	Dichotomous	1. Yes<br>2. No
c33	Drunk alcohol in the past 1 year	Dichotomous	1. Yes<br>2. No
c34	Stop drinking for health reason	Dichotomous	1. Yes<br>2. No
c35	Frequency of drinking in the past 1 year	Categorical	1. > 6 times/week<br>2. 3-5 days/week<br>3. 1-2 days/week<br>4. 1-3 times/month<br>5. < 1 time/month
c36	Remember the number of alcohol units each time on average	Dichotomous	1. Yes<br>2. No
c36a	Number of alcohol units each time on average	Dichotomous	
c37	Doing exercise in the past 1 year	Dichotomous	1. Yes<br>2. No
c38	Frequency of doing exercise	Categorical	1. Every day<br>2. 5-6 days/week<br>3. 3-4 days/week<br>4. 1-2 days/week
c39	Duration of time of doing exercise per day on average	Continuous	
c40	Low salt diet	Dichotomous	1. Yes<br>2. No
c41	Limit processed foods	Dichotomous	1. Yes

Variable code	Variable label	Variable type	Variable value label
			2. No
c42	Limit extra salt while eating	Dichotomous	1. Yes<br>2. No
c43	Limit eating salty food	Dichotomous	1. Yes<br>2. No
c45	Current treatment for hypertension	Categorical	1. No treatment<br>2. Self-medicating without prescription<br>3. Self-medicating with a previous prescription<br>4. Being monitored and treated at a health facility
c46	Frequency of regular check-up at health facility	Categorical	1. Every month<br>2. 2-3 months/time<br>3. > 4 months/time
c47	Taking anti-hypertensive medicines	Dichotomous	1. Yes<br>2. No
c48	Forgot to take anti-hypertensive medicines	Dichotomous	1. Yes<br>2. No
c49	Were there any day of not taking anti- hypertensive medicines in the past 2 weeks	Dichotomous	1. Yes<br>2. No
c50	Stopped taking medications when you felt worse	Dichotomous	1. Yes<br>2. No
c51	Forgot to bring medications when travelling or leaving home	Dichotomous	1. Yes<br>2. No
c52	Take all medicines yesterday	Dichotomous	1. Yes<br>2. No
c53	Stopped taking medications when you feel like health condition is under control	Dichotomous	1. Yes<br>2. No
c54	Taking medications daily is inconvenient	Dichotomous	1. Yes<br>2. No
c55	How often do you have difficulty remembering to take all your medicines every day?	Categorical	1. Never<br>2. Rarely<br>3. Sometimes<br>4. Usually<br>5. All the time
**D. THE ACCESSIBILITY AND UTILIZATION OF HEALTH SERVICES**			
d1	Facility for hypertension treatment	Categorical	1. Commune health station<br>2. District health center<br>3. Provincial hospital<br>4. Private health facility

Variable code	Variable label	Variable type	Variable value label
			5. Other, specify
d1_khac	Facility for hypertension treatment (other, specify)	Text	
d2	Reason for not getting treatment for hypertension at surveyed CHS	Categorical	1. It is not the primary care facility following health insurance enrolment<br>2. Have other co-morbidity<br>3. Unsatisfied with attitude of CHS staffs<br>4. CHS staff do not examine carefully<br>5. CHS staff do not give medical advice or counsel patients properly<br>6. This CHS are small/poorly organized/untidy<br>7. This CHS lacks medical equipment<br>8. CHS often lacks medicines<br>9. The range of health services are limited and do not meet my demand<br>10. Have to pay extra money<br>11. Other, specify
d2_khac	Reason for not getting treatment for hypertension at surveyed CHS (other, specify)	Text	
d3	Reason for choosing CHSs to get periodic treatment for hypertension	Categorical	1. I registered this CHS as my primary care facility under my health insurance enrolment<br>2. I have a mild disease/Only get hypertension<br>3. Satisfied with the staff's attitude<br>4. Health workers give careful and complete consultation<br>5. Health workers examine carefully<br>6. The condition of disease is improved<br>7. Satisfied with facilities of CHSs<br>8. CHSs provide enough medicine every month<br>9. CHSs have right for my treatment

Variable code	Variable label	Variable type	Variable value label
			10. No extra cost for examination and treatment<br>11. Near house<br>12. Do not have to wait for a long time<br>13. To be referred (friends, relatives, health workers,…)<br>14. Other, specify
d3_khac	Reason for choosing CHSs to get periodic treatment for hypertension (other, specify)	Text	
d3a	Number of visits to CHS for examination and treatment in the past 12 months	Continuous	
d4	Services received at CHS	Categorical	1. Measure height, weight, waist circumference, hip circumference, ...<br>2. Measure blood pressure level<br>3. General examination<br>4. Ask about the patient's symptoms<br>5. Ask about risk factors<br>6. Personal counselling<br>7. Group consultation<br>8. Received document/information leaflet<br>9. Check personal medical examination/self-monitoring book<br>10. Provide anti-hypertensive drugs<br>11. Tests<br>12. Ultrasound, ECG<br>13. Other, specify
d4_khac	Services which patients received at CHS (other, specify)	Text	
d51	Medicines were prescribed	Text	
d52	Contents of the counselling sections	Categorical	1. The level of hypertension<br>2. Complications of hypertension<br>3. How to take anti-hypertensive drugs<br>4. Drug side effects<br>5. Exercise regime<br>6. Restrict the use of stimulants (smoking, drinking alcohol, ...)

Variable code	Variable label	Variable type	Variable value label
			7. Diet rich in green vegetables and fruits<br>8. Low salt diet<br>9. The role and significance of adherence (medication and lifestyle changes)<br>10. Target blood pressure level<br>11. Self-monitoring of blood pressure<br>12. How to take notes and use notebooks to monitor treatment<br>13. Other, specify
d52_khac	Contents of the counselling sections (other, specify)	Text	
d7	Buy additional medicine	Dichotomous	1. Yes<br>2. No
d7a	Types of extra medicines	Categorical	1. Anti-hypertensive drugs<br>2. Other medicines
d7b	Reason for buying extra medicines	Categorical	1. CHS's drugs not covered by health insurance<br>2. Drugs prescribed were not covered by health insurance<br>3. Do not trust the quality of drugs covered by health insurance<br>4. Other, specify
d7b	Reason for buying extra medicines (other, specify)	Text	
d8	Level of satisfaction with services related to hypertension treatment at CHS/DHC (1-10)	Continuous	
d9	Difficulties while using hypertension treatment services at the CHS/DHC	Categorical	1. Far from home/No one to take or pick up<br>2. Health workers are not enthusiastic<br>3. Insufficient counseling<br>4. No careful examination<br>5. Low qualifications of health workers<br>6. Lack of medical staff<br>7. Lack of necessary equipment<br>8. Unsatisfied with the infrastructure<br>9. Lack of drugs in quantity<br>10. Poor range of drugs

Variable code	Variable label	Variable type	Variable value label
			11. CHSs does not provide services such as testing, ultrasound, etc.<br>12. Pay extra cost for examination and treatment<br>13. The condition has not improved<br>14. Long waiting time<br>15. Other, specify
d9	Difficulties while using hypertension treatment services at the CHS/DHC (other, specify)	Text	
**E. FAMILY AND SOCIETY SUPPORT**			
e1	Sources of information about hypertension	Categorical	1. Village health workers<br>2. CHS staff<br>3. Relatives<br>4. Friends<br>5. ISHCs<br>6. Local community groups and community<br>7. Internet<br>8. Books, newspapers, magazines<br>9. TV<br>10. Speakers, radios<br>11. Other, specify
e1_khac	Sources of information about hypertension (other, specify)	Text	
e2	Received any support in hypertension treatment from family members	Dichotomous	1. Yes<br>2. No
e21	Kinds of support	Categorical	1. Take care of<br>2. Remind/encourage to take medicine<br>3. Remind/encourage to maintain a healthy lifestyle<br>4. Support/take to CHS<br>5. Other, specify
e21_khac	Kinds of support (other, specify)	Text	
e3	Have groups/organizations/clubs related to hypertension in local area	Dichotomous	1. Yes<br>2. No
e31	Have participated in these groups	Dichotomous	1. Yes<br>2. No
e32	Names of groups/organizations/clubs related to hypertension which patients participated	Text	

Variable code	Variable label	Variable type	Variable value label
e33	Be supported/encouraged in adherence to treatment from these groups/organizations/clubs	Dichotomous	1. Yes<br>2. No
e34	Kinds of support	Categorical	1. Provide information about hypertension<br>2. Provide information about hypertension treatment<br>3. Get periodic health examination<br>4. Support/remind/encourage for treatment and adherence<br>5. Referred to health facilities<br>6. Other, specify
e34_khac	Kinds of support (other, specify)	Text	
e35	Kinds of mobile phone currently use	Categorical	1. Do not use mobile phone<br>2. Smartphone<br>3. Basic cellphone<br>4. Other, specify
e35_khac	Kinds of mobile phone currently use (other, specify)	Text	
e36	Functions which patients are proficient	Categorical	1. Normal listen and call<br>2. Video call<br>3. Texting<br>4. Send/receive email<br>5. Listen to music/take pictures<br>6. Use the internet to read and search information<br>7. Using phone's apps<br>8. Other, specify
e36_khac	Functions which patients are proficient (other, specify)	Text	
**F. QUALITY OF LIFE**			
f1	Difficulty on movement	Categorical	1. No problem<br>2. A bit of trouble<br>3. Moderate difficulty<br>4. A lot of difficulties<br>5. Only stay in bed
f2	Difficulty on self-care	Categorical	1. No problem<br>2. A bit of trouble<br>3. Moderate difficulty<br>4. A lot of difficulties<br>5. Cannot do by self
f3	Difficulty on routine activities	Categorical	1. No problem<br>2. A bit of trouble<br>3. Moderate difficulty

Variable code	Variable label	Variable type	Variable value label
			4. A lot of difficulties<br>5. Cannot perform routine activities
f4	Pain/discomfort	Categorical	1. No pain or discomfort<br>2. A little pain<br>3. Moderate pain or discomfor<br>4. Hurt or slightly uncomfortable<br>5. In pain or very uncomfortable
f5	Anxiety	Categorical	1. Not worried or not melancholy<br>2. A bit worried or melancholy<br>3. Worried or melancholy at medium level<br>4. Worried or melancholy<br>5. Extremely worried or melancholy
**G. PAYMENTS NOT COVERED BY HEALTH INSURANCE FOR MEDICAL SERVICES**			
g1	Visit a medical facility for inpatient or outpatient treatment to treat hypertension, diabetes, related diseases in the past 3 months	Dichotomous	1. Yes<br>2. No
g2	Number of visits in the past 3 months	Continuous	
id_ho1	Patient ID	Continuous	
lan_ngoai_tru	Order of outpatient visit	Continuous	
op_id	Outpatient ID	Continuous	
g3	Health facility for outpatient visit	Categorical	1. CHS<br>2. Local DHC<br>3. Local District Hospital<br>4. Other DHC<br>5. Other District Hospital<br>6. Provincial/city preventive medicine center<br>7. Provincial/city hospital<br>8. Central Hospital<br>9. Private Hospital<br>10. Private Clinic<br>11. Home visit doctor<br>12. Healers<br>98. Other, specify<br>99. Unknown/No answer
g3_khac	Health facility for outpatient visit (other, specify)	Text	

Variable code	Variable label	Variable type	Variable value label
g4	Reasons for a visit	Categorical	1. Monthly check-ups (hypertension/diabetes)<br>2. Examination due to high blood pressure<br>3. Examination due to low blood sugar<br>4. Examination due to increased blood sugar<br>5. Other, specify
g4_khac	Reasons for a visit (other, specify)	Text	
g5a	Any out of pocket payment	Dichotomous	1. Yes<br>2. No
g5	Direct medical cost for OP	Continuous	
g6	Direct non-medical cost for OP	Continuous	
g7	Other informal payment for OP	Continuous	
g8	Total payment for outpatient visit	Continuous	
g9	Use health insurance card this time	Dichotomous	1. Yes<br>2. No
g10	Amount to be paid by health insurance	Continuous	
g11	Does the household afford to pay for outpatient care?	Categorical	1. Yes, enough money<br>2. Yes, not enough money<br>3. No money
g12	If no, how much money that household could not afford for outpatient care?	Continuous	
g13	How to pay for the sum that household could not afford	Categorical	1. Sell properties<br>2. Sell land<br>3. Loan with no interest<br>4. Loan with interest<br>5. Withdrawal of saving money/stake/debt collection/loan clubs<br>6. Ignore the treatment<br>98. Other (specify)<br>99. Unknown
g13_khac	Other coping methods for outpatient care (other, specify)	Text	
g14	The patient walks on their own or go with relatives?	Dichotomous	1. Walks on their own<br>2. Go with relatives
**H. HOSPITALIZATION**			
h1	Hospitalization due to hypertension, diabetes, related diseases in the past 3 months	Dichotomous	1. Yes<br>2. No

Variable code	Variable label	Variable type	Variable value label
h2	Number of hospital admission in the past 3 months	Continuous	
id_ho2	Patient ID	Continuous	
lan_noi_tru	Order of inpatient visit	Continuous	
ip_id	Inpatient ID	Continuous	
h3	Health facility for hospitalization	Categorical	1. CHS<br>2. DHC where you live<br>3. District Hospital where you live<br>4. Other DHC<br>5. Other District Hospital<br>6. Provincial/city preventive medicine center<br>7. Provincial/city hospital<br>8. Central Hospital<br>9. Private Hospital<br>98. Other, specify<br>99. Unknown/No answer
h3_khac	Health facility for hospitalization (other, specify)	Text	
h4	Reason for hospitalization	Categorical	1. High blood pressure<br>2. High blood glocuse<br>3. Low blood glocuse<br>4. Stroke<br>5. Heart complications<br>6. Eye Complications<br>7. Kidney complications<br>8. Other, specify
h4_khac	Reason of hospitalization (other, specify)	Text	
h4a	Number of inpatient (IP) days	Continuous	
h5a	Out of pocket payment for IP	Dichotomous	1. Yes<br>2. No
h5	Direct medical cost for IP	Continuous	
h6	Direct non-medical cost for IP	Continuous	
h7	Other informal payment for IP	Continuous	
h8	Total payment for IP care	Continuous	
h9	Use health insurance card this time	Dichotomous	1. Yes<br>2. No
h10	Amount to be paid by health insurance	Continuous	
h11	Does the household afford to pay for IP care?	Categorical	1. Yes, enough money<br>2. Yes, not enough money<br>3. No money

Variable code	Variable label	Variable type	Variable value label
h12	If no, how much money that household could not afford for IP care?	Continuous	
h13	How to pay for the sum that household could not afford	Categorical	1. Sell properties<br>2. Sell land<br>3. Loan with no interest<br>4. Loan with interest<br>5. Withdrawal of saving money/stake/debt collection/loan clubs<br>6. Ignore the treatment<br>98. Other (specify)<br>99. Unknown
h13_khac	Other coping method for IP care (other, specify)	Text	
**K. SELF-TREATMENT**			
k1	Self-treatment in the past 3 months	Dichotomous	1. Yes<br>2. No
k2	Number of times of self-treatment in the past 3 months	Continuous	
k3	Total cost for self-treatment	Continuous	
**M. IMPACT OF COVID-19**			
m1	Main sources that used to update information about Covid-19	Categorical	1. Newspapers<br>2. Television<br>3. Radio<br>4. Government websites<br>5. Local speakers<br>6. Facebook/Other Social Networks<br>7. Community groups<br>8. Health staff<br>9. Local communication sessions<br>10. Other (specify)<br>11. Unknown
fm_khac	Main sources that used to update information about Covid-19 (other, specify)	Text	
m2	Easy or difficult to identify reliable information about Covid-19	Categorical	1. Very easy<br>2. Somewhat easy<br>3. Somewhat difficult<br>4. Very difficult
m3	Self-assessment of risk of virus infection	Categorical	1. No risk<br>2. Low risk<br>3. High risk<br>4. Very high risk

Variable code	Variable label	Variable type	Variable value label
m4	Self-assessment of severe course associated with virus infection	Categorical	1. No risk<br>2. Low risk<br>3. High risk<br>4. Very high risk
m5	People with diabetes, hypertension, CVDs have a higher risk of COVID-19 than others	Categorical	1. Yes<br>2. No<br>3. Unknown
m6	People with diabetes, hypertension, and CVDs have a higher risk of getting complication after infection	Categorical	1. Yes<br>2. No<br>3. Unknown
f7a	Covid impact_work and/or school participation	Categorical	1. Better<br>2. Unchanged<br>3. Worse
f7b	Covid impact_food security	Categorical	1. Better<br>2. Unchanged<br>3. Worse
f7c	Covid impact_physical health	Categorical	1. Better<br>2. Unchanged<br>3. Worse
f7d	Covid impact_mental health	Categorical	1. Better<br>2. Unchanged<br>3. Worse
f7e	Covid impact_availability of medicine for your conditions	Categorical	1. Better<br>2. Unchanged<br>3. Worse
f7f	Covid impact_income	Categorical	1. Better<br>2. Unchanged<br>3. Worse
f7g	Covid impact_general expenditure	Categorical	1. Better<br>2. Unchanged<br>3. Worse
f7h	Covid impact_health expenditure	Categorical	1. Better<br>2. Unchanged<br>3. Worse
f8a	Get vaccination if the Ministry of Health advises people	Dichotomous	1. Agree<br>2. Not agree
f8b	Get vaccination if relatives/friends advise me	Dichotomous	1. Agree<br>2. Not agree
f8c	Get vaccination if it is free	Dichotomous	1. Agree<br>2. Not agree
